# Electrical Conduction Behavior of High-Performance Microcellular Nanocomposites Made of Graphene Nanoplatelet-Filled Polysulfone

**DOI:** 10.3390/nano10122425

**Published:** 2020-12-04

**Authors:** Hooman Abbasi, Marcelo Antunes, José Ignacio Velasco

**Affiliations:** Department of Materials Science and Engineering, Poly2 Group, Technical University of Catalonia (UPC BarcelonaTech), ESEIAAT, C/Colom 11, E-08222 Terrassa, Spain; hooman.abbasi@upc.edu (H.A.); marcelo.antunes@upc.edu (M.A.)

**Keywords:** cellular nanocomposite, electrical conductivity, graphene nanoplatelets, polysulfone

## Abstract

Graphene nanoplatelet (GnP)-filled polysulfone (PSU) cellular nanocomposites, prepared by two different methods—namely, water vapor-induced phase separation (WVIPS) and supercritical CO_2_ dissolution (scCO_2_) foaming—were produced with a range of densities from 0.4 to 0.6 g/cm^3^ and characterized in terms of their structure and electrical conduction behavior. The GnP content was varied from 0 to 10 wt%. The electrical conductivity values were increased with the amount of GnP for the three different studied foam series. The highest values were found for the microcellular nanocomposites prepared by the WVIPS method, reaching as high as 8.17 × 10^−2^ S/m for 10 wt% GnP. The variation trend of the electrical conductivity for each series was analyzed by applying both the percolation and the tunneling models. Comparatively, the tunneling model showed a better fitting in the prediction of the electrical conductivity. The preparation technique of the cellular nanocomposite affected the resultant cellular structure of the nanocomposite and, as a result, the porosity or gas volume fraction (*V_g_*). A higher porosity resulted in a higher electrical conductivity, with the lightest foams being prepared by the WVIPS method, showing electrical conductivities two orders of magnitude higher than the equivalent foams prepared by the scCO_2_ dissolution technique.

## 1. Introduction

Multifunctional polymer nanocomposites have been the center of attention in the scientific community for the last decade due to their capabilities in providing combined thermal, acoustic mechanical, and weight reduction properties, among others [1]. Various studies have considered the creation of cellular materials using high-performance thermoplastics for cutting-edge industries, such as aerospace, electronics, or telecommunications. Among high-performance thermoplastics, polysulfone (PSU) has been considered due to its high thermal stability and inherent fire resistance; its excellent mechanical properties—namely, high strength and toughness; and its good environmental stress-crack resistance [2,3]. Additionally, its high resistance to gamma and e-beam radiation due to its high aromatic content, alongside its good hydrolytic stability, makes PSU a suitable candidate for components requiring sterilization [4].

PSU-based cellular nanocomposites prepared by CO_2_ dissolution batch foaming [5,6,7,8,9]; continuous extrusion chemical foaming; and solution phase separation—in this last case, used for preparing PSU-based membranes [10], have been studied vastly. Various studies have been conducted on PSU cellular nanocomposites and membranes with carbon-based nanoparticles [11,12,13,14]; however, the study of such cellular nanocomposites with respect to their electrical conductivity has not yet been explored. Sánchez et al. [15,16] presented promising results regarding the fabrication of carbon nanotube and polysulfone thick-film screen-printed electrodes for electrochemical enzyme biosensor and immune-sensor applications.

Carbon-based nanofillers such as graphene nanoplatelets (GnP) and carbon nanotubes (CNT) have attracted a great deal of interest, as they have been shown to enable the fabrication of polymer nanocomposites with enhanced transport properties, counteracting some limitations of electrically conductive polymers for advanced sectors [17]. Additionally, they can provide polymer nanocomposites with improved mechanical properties and multifunctionality [18].

Recent focus on creating cellular nanocomposites containing conductive nanoparticles has led to the development of novel materials with improved specific properties—for instance, in terms of electrical and thermal conductivities. Regarding this matter, studies microcellular nanocomposites based on polyetherimide filled with carbon-based nanofillers have been recently published [19,20,21,22,23]. In this sense, we have previously reported the thermal conductivity behavior of PSU-based cellular nanocomposites containing graphene nanoplatelets [24].

In the present paper, the preparation of GnP–PSU microcellular nanocomposites has been carried out using two different processing techniques—namely, water vapor-induced phase separation (WVIPS) and one-step supercritical CO_2_ (scCO_2_) dissolution. The dispersion of the GnP nanoparticles promoted during the solution mixing and phase inversion step of WVIPS process, as well as during the melt-mixing and subsequent scCO_2_ dissolution and expansion steps in the gas dissolution process, could result in an enhanced electrical conductivity at a low GnP concentration, enabling the use of these novel nanocomposites, for instance, in electrostatic discharge (ESD) or electromagnetic interference (EMI) shielding applications.

## 2. Materials and Methods

### 2.1. Materials and Foaming

Polysulfone, in the form of pellets, with the commercial name of UDEL P-1700, a density of 1.24 g/cm^3^, and a glass transition temperature of 185 °C, was used in the present investigation. The graphene nanoplatelets (xGnP-Grade M15) used in this study were purchased from XG Sciences Inc. (Lansing, MI, USA), with a reported thickness of 6–8 nm; an average platelet diameter of 15 µm; a surface area of 120–150 m^2^/g; a density of 2.2 g/cm^3^; and an electrical conductivity of 10^7^ and 10^2^ S/m, measured parallel and perpendicular to their surface, respectively. N-methyl pyrrolidone (NMP) was obtained from Panreac Química SA (Barcelona, Spain) with a purity of 99% and boiling and flash points of 202 and 95 °C, respectively.

Both the foaming processes used in this study are explained in detail in a previously published article [24]. The resultant microcellular nanocomposites could be categorized in three different series. The first and second series were prepared using the WVIPS method with two different concentrations of the initial PSU solution, in both cases containing between 0 and 10 wt% GnP, the first one using 15 wt% PSU in NMP, resulting in an average density of 0.47 g/cm^3^, and the second one using 25 wt% PSU in NMP (average density 0.52 g/cm^3^). From here on, these microcellular nanocomposites will be referred to as “15PSU x” and “25PSU x”, respectively, where x represents the amount of graphene in weight percentage. The third series of microcellular nanocomposites were prepared by a one-step scCO_2_ dissolution, resulting in microcellular nanocomposites with an average density of 0.50 g/cm^3^ containing 0–2 wt% of GnP. These samples were coded as “PSUCO_2_ x”.

### 2.2. Testing Procedure

The density values of the microcellular nanocomposites were measured by the application of the ISO-845 standard method. The gas volume percentage (*V_g_*) values were calculated from the density of the foam and respective unfoamed material according to the following expression:

(1) $$V_{g}=\left(1-\frac{\delta }{\delta_{s}}\right)\times 100$$

where δ and δ_s_ are the density of the foamed and unfoamed materials, respectively (δ/δ_s_ is the so-called relative density).

The analysis of the cellular structure was performed using a scanning electron microscope (SEM), particularly a JEOL JSM-5610 (Tokyo, Japan), by applying a 10 kV voltage at a working distance of 40 mm to samples that had been brittle-fractured in liquid nitrogen. The samples were later coated with a thin layer of gold by sputter deposition, using a sputter coater (BAL-TEC SCD005; Los Angeles, CA, USA) under an argon atmosphere.

X-ray diffraction (XRD) was used to evaluate the effectiveness of the GnP nanoparticle dispersion by analyzing the characteristic (002) diffraction plane of GnP and the possible crystallinity of PSU, using a PANalytical diffractometer (Almelo, The Netherlands) operating with CuK α (λ = 0.154 nm) at 40 kV and 40 mA. The scanning range was from 2 to 60° using a scan step of 0.033°.

The electrical conductivity measurements were performed on 20 mm × 20 mm × 1 mm samples directly cut from the prepared microcellular nanocomposites using a HP 4140B pA meter and dc voltage source with a two-probe set. A thin layer of colloidal silver conductive paint was used to cover the surfaces of the samples in contact with the copper electrode pads, which had an electrical resistance between 0.01 and 0.1 Ω/cm^2^. A direct current voltage was applied with a range of 0–5 V, with a voltage step of 0.05 V, a hold time of 10 s, and a step delay time of 5 s. The electrical conductivity (σ in S/m) was calculated according to:

(2) $$\sigma =\frac{1}{\rho_{v}}$$

and

(3) $$\rho_{v}=\frac{RA_{E.C}}{d}$$

where $\rho_{v}$ (in Ω·m) represents the electrical volume resistivity, *R* is the electrical resistance of the sample in Ω, *A_E.C_* is the area of the surface in m^2^, and *d* is the distance between electrodes (in m). The following expressions (Equations (4)–(6)) were used in order to normalize the measured values of electrical conductivity, regarding the gas volume fraction of the microcellular nanocomposites, which could influence the effective contact surface area between the sample and the electrodes. The average cell size and the cell density of the microcellular nanocomposites were used in order to obtain the corrected value of electrical conductivity (σ*), taking into account variations in the effective surface area as follows:

(4) $$\sigma *=\frac{d}{R\left(A_{nc}+A_{ch}\right)}$$

where *A_nc_* (no-cell area) is defined as the *A_E.C_* with the cell section area excluded, and the area of the cell hemisphere (*A_ch_*) was calculated using:

(5) $$A_{ch}=\left(\frac{n}{A}\right)A_{E.C}\left(2\pi \frac{\psi^{2}}{4}\right)$$

Therefore,

(6) $$A_{nc}+A_{ch}=A_{E.C}+\left(\left(\frac{n}{A}\right)A_{E.C}\left(\pi \frac{\psi^{2}}{4}\right)\right)$$

The values of *n*, *A*, and ψ were obtained from the analyzed SEM micrographs [24] and represent the number of cells, the corresponding area of the micrograph at ×300 magnification in m^2^, and the average cell size, respectively.

## 3. Results

### 3.1. X-ray Diffraction Analysis

The density and the intensity values of the XRD peak at 2θ = 26.5°, corresponding to the (002) crystallographic plane of graphene nanoplatelets, for all the microcellular nanocomposites, are presented in Table 1.

The PSU-based microcellular nanocomposites, prepared using both scCO_2_ dissolution and WVIPS, presented density values between 0.435 and 0.568 g/cm^3^, with evidence of variations related to the PSU and solvent proportions in microcellular nanocomposites prepared via WVIPS and PSU and GnP concentrations (in all series). The microcellular nanocomposites prepared using the WVIPS method showed a decreasing trend, in terms of density, by reducing the amount of polymer in the solution from 25 to 15 wt%. Additionally, the increase in GnP concentration resulted in a significant rise in density for all the samples. In the series prepared by scCO_2_ dissolution, the density value increased 25% by increasing the GnPs’ weight percentage from 0.1 to 2.0. A similar trend was observed in samples prepared by WVIPS, with the density increasing more than 13% and 42% in 15PSU and 25PSU, respectively.

**Figure 1:**
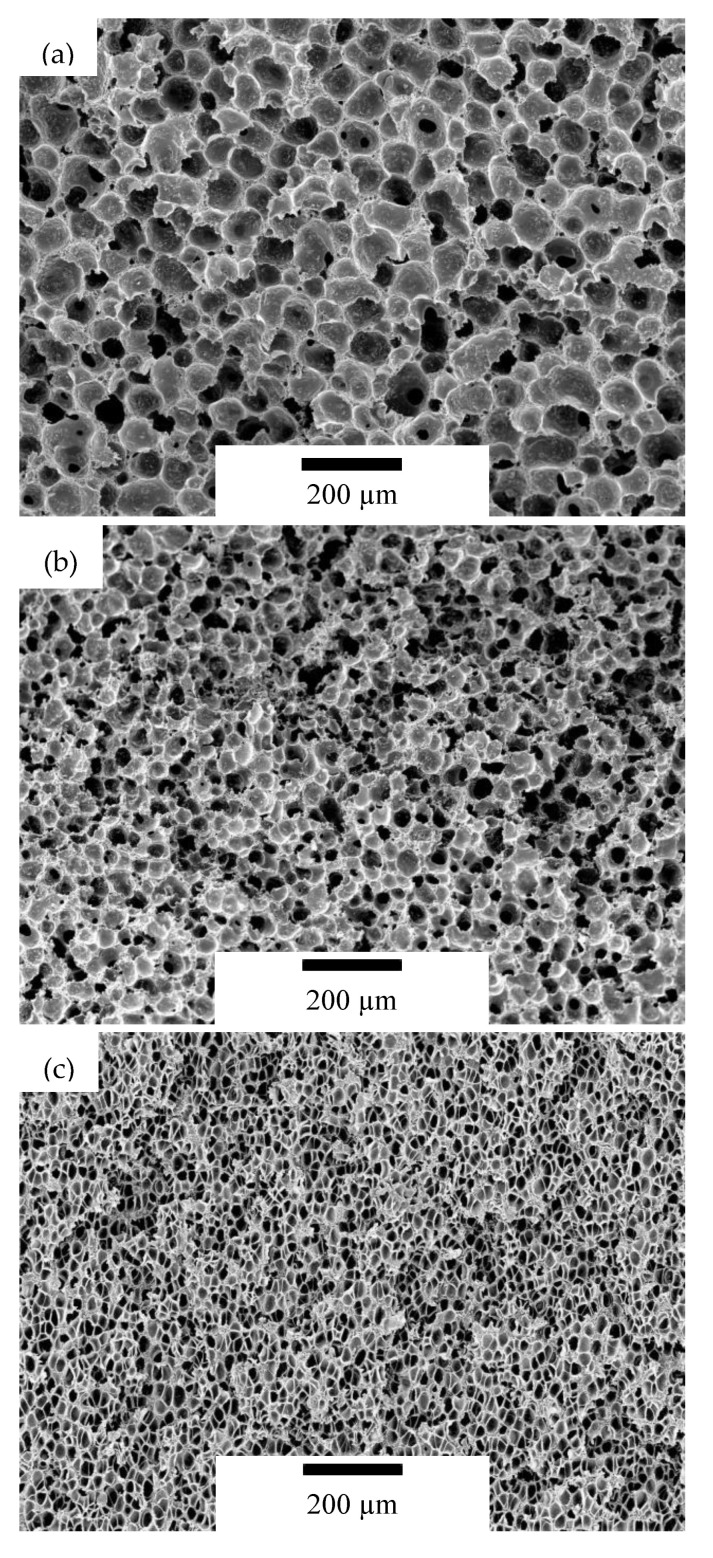
Micrographs at ×95 magnification showing the cellular structure of microcellular nanocomposites containing 2 wt% GnP: (**a**) 15PSU 2, (**b**) 25PSU 2, and (**c**) PSUCO_2_ 2 samples.

The cellular structure of microcellular nanocomposites containing 2 wt% GnP is presented in Figure 1. The samples of the 15PSU series presented a closed-cell structure with an average cell size of around 54 µm, with the exception of the sample containing 10 wt% GnP, which showed an open interconnected foam structure. A similar open or interconnected structure also appeared in 25PSU samples containing 5 and 10 wt% GnP. Other PSU–GnP microcellular nanocomposites in this series presented an average cell size around 28 µm. All of the samples prepared by scCO_2_ dissolution presented a closed-cell structure, with an average cell size of 15 µm.

**Figure 2:**
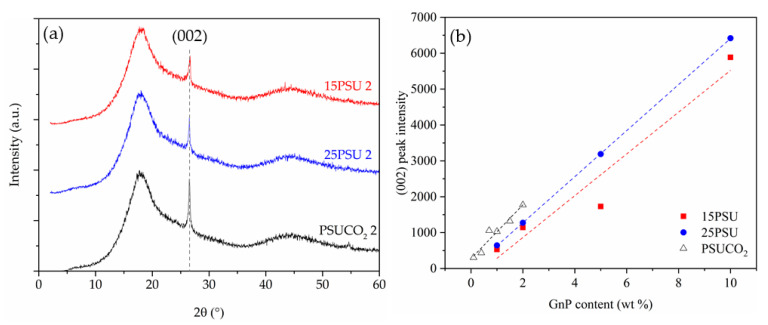
(**a**) XRD spectra of microcellular nanocomposites containing 2 wt% GnP with the presence of a characteristic (002) diffraction plane of GnP at 2θ = 26.5 and (**b**) the normalized intensity value of the (002) diffraction plane of the GnP peak for PSU and PSU–GnP microcellular nanocomposites. The peak intensity was normalized by dividing the intensity by the density and presented using a linear fit with respect to the concentration of GnP.

As can be seen in Figure 2, the intensity of the characteristic (002) diffraction plane of GnP found at 2θ = 26.5° suggests a possible effect of various foaming and mixing methods on the dispersion level of GnP throughout the cellular structure. The results show that solvent mixing favors the dispersion level of graphene nanoplatelets, which could be related to the lower viscosity of the mixing medium when compared to the melt-mixing process. Similarly, the changes in the peak intensity of microcellular nanocomposites prepared by WVIPS indicate that variations in the foaming process, particularly by decreasing the viscosity of the solution (25% PSU vs. 15% PSU in NMP), could result in a better dispersion of graphene stacks, especially noticeable at a higher nanofiller concentration (5 and 10 wt% GnP).

### 3.2. Electrical Conductivity

The electrical conductivity of the PSU–GnP microcellular nanocomposites showed a general rise with the increasing GnP content, as expected, owing to the inherently high electrical conductivity of GnP, particularly presenting higher increases in foams prepared by WVIPS. The values of density, *V_g_*, GnP weight and volume percentages, electrical conductivity, and corresponding corrected values are presented in Table 2.

**Figure 3:**
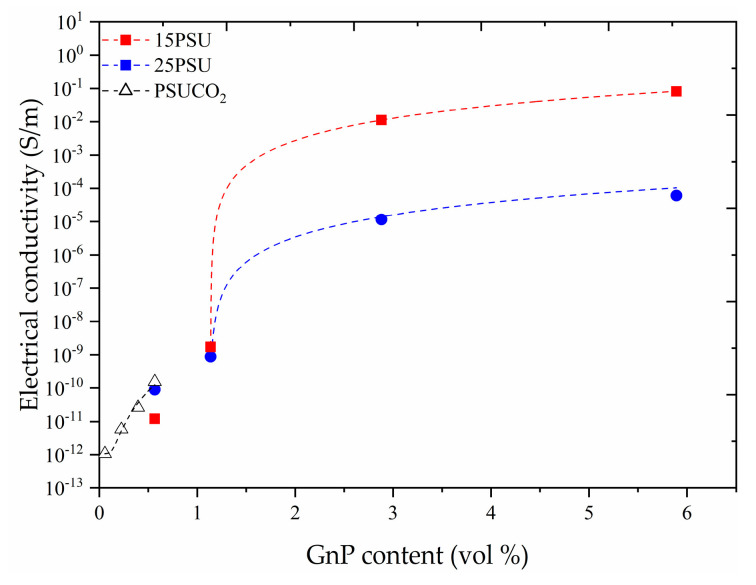
Evolution of the electrical conductivity with GnP content for the three foam series representing a power law fit.

As can be seen in Figure 3, the increasing trend in electrical conductivity slows down as the concentration of GnP increases, demonstrating a possible limitation in terms of GnP dispersion at higher concentrations. 

**Figure 4:**
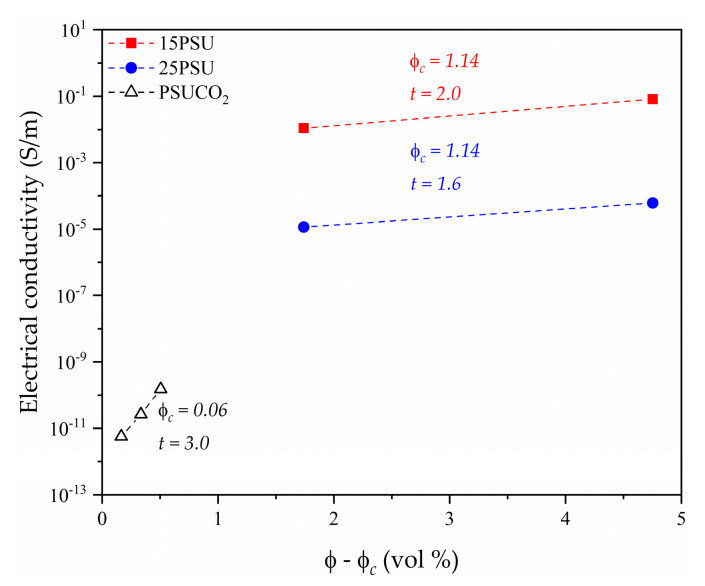
Evolution of the electrical conductivity of PSU–GnP nanocomposites with ϕ–$\phi_{c}$ including percolation fitting curves and the values of the percolation threshold and the percolation exponent.

As can be seen in Figure 3, the electrical conductivity increased for all the microcellular nanocomposites with the addition of GnP, reaching values as high as 8.2 × 10^−2^ S/m with 2.7 vol% (10 wt%) graphene nanoplatelets in 15PSU. The percolation model is commonly used to depict the electrical conductivity of nanocomposites as a function of the amount of conductive particles above a certain critical point, based on the physical contact between them, using:

(7) $$\sigma \propto {\left(\phi -\phi_{c}\right)}^{t}$$

where ϕ is the volume fraction of the conductive particles, $\phi_{c}$ the percolation threshold, and *t* is the percolation exponent [19,25,26,27,28]. However, this model is sensitive to the concentration range of GnP and does not consider that nanocomposites could already present a level of electrical conduction at GnP concentrations below the critical value, as can be seen in Figure 4. Therefore, a tunnel conduction mechanism was suggested as the dominant model in the GnP concentration range used in this study, since it has been previously demonstrated to be a better fitting model for cellular materials containing conductive fillers [20,21,23,29] before the formation of a continuous conductive particle network.

**Figure 5:**
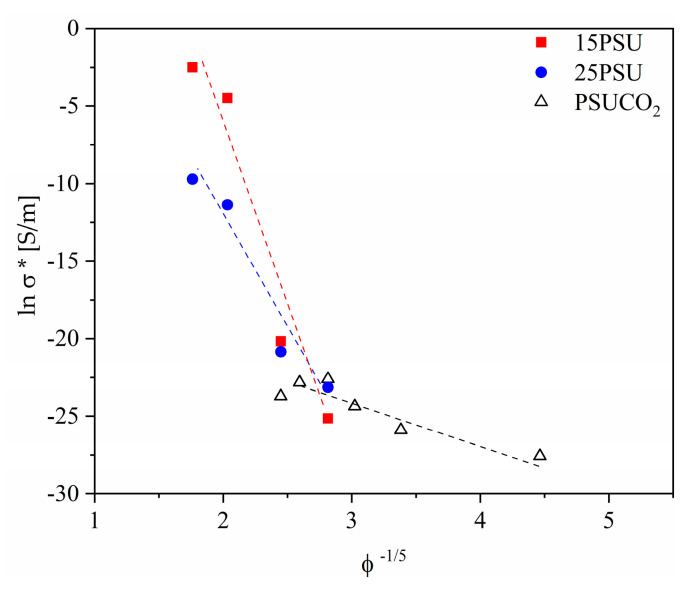
Representation of the fitting results of the electrical conductivity versus ϕ^−1/5^, demonstrating the tunnel conduction characteristics of a 3D random particle distribution system formed by conductive GnP stacks.

Considering a tunnel conduction mechanism, the electrical conductivity could be predicted using:

(8) σ∝exp(−Ad)

where *A* and *d* represent the tunnel parameter and distance, respectively [30]. The results suggest that the tunneling distance remains a slightly closer fit to that of a three-dimensional random particle distribution as opposed to a two-dimensional network, with minimal differences. Figure 5 demonstrates the best fitting of the corrected electrical conductivity as a function of ϕ^−1/5^ (with the exponent representing the geometry factor). 

**Figure 6:**
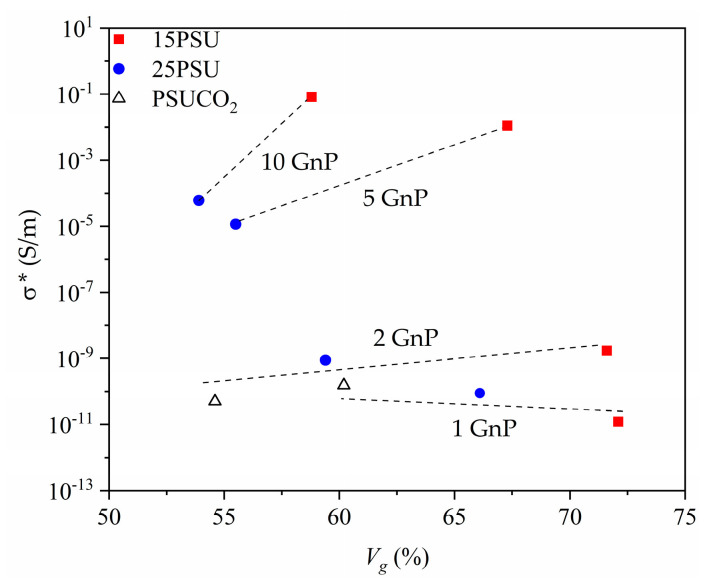
Effect of the gas volume percentage on the electrical conductivity of PSU–GnP microcellular nanocomposites represented by linear fits.

Interestingly, the foaming process seemed to have a positive influence on the electrical properties of the prepared microcellular nanocomposites, as can be seen in Figure 6, with the microcellular nanocomposites having a lower density (higher gas volume fraction) and showing higher values of electrical conductivity (similar to the results published in our previous study [23]). 

The microcellular nanocomposites obtained in this study display electrical conductivities that enable their use in applications such as ESD or EMI shielding. Samples prepared by scCO_2_ dissolution foaming could be used in ESD applications, since the required electrical resistivity range for their implementation is between 10^12^ and 10^5^ Ω [31]. Since the EMI shielding capability of materials is closely related to their electrical conductivity [32,33], as materials require an electrical resistance value below 10^5^ Ω [31], the samples prepared by the WVIPS method (15PSU and 25PSU) could be suitable candidates for this application, with values as high as 10^−2^ S/m and volume percentages of graphene as low as 0.94%.

## 4. Further Discussion

Regarding the electrical conductivity of the prepared microcellular nanocomposites, the influence of the conductive filler distribution and dispersion on the eventual conductivity of nanocomposites is widely known [34,35,36,37]. In previous works on polyetherimide (PEI) [19,21,23], we have shown that dispersion processes, such as the application of ultrasonication and melt-mixing, could improve the electrical conductivity of these nanocomposites. Additionally, the mentioned results indicated that further electrical conductivity improvements could be achieved through foaming by decreasing the interparticle distance between conductive nanoparticles distributed throughout the cell walls. The electrical conductivity values suggest a good dispersion of the nanoparticles after melt-mixing at low concentrations of GnP; however, for higher nanofiller contents, the combination of ultrasonication and solution mixing showed a higher efficiency in providing a desirable dispersion level.

In the case of the microcellular nanocomposites prepared by scCO_2_ dissolution foaming, a clear change in trend appeared after 1 wt% GnP, while in the case of the microcellular nanocomposites prepared by WVIPS it was observed at 5 wt% GnP, showing that the WVIPS was more effective in providing a better GnP dispersion, which could be related to the lower viscosity of the medium in this method (NMP-based dissolution), when compared to that of melt-compounding required for preparing PSUCO_2_ samples.

A similar study involving nanocomposites of thermoplastic polyurethane and graphene oxide [34] presented a comparison between various dispersion methods—namely, solvent mixing and melt-compounding. The results suggested that an improved dispersion was achieved in solvent mixing when compared to melt-compounding. This could be due to the higher viscosity of the matrix in the latter method.

Regarding the fitting tunnel conduction mechanism, similar results were observed for the fitting curve with ϕ^−1/3^, which could be a sign of network formation in both three-dimensional nanoparticles with the assistance of a two-dimensional network formed by exfoliated nanoparticles, as suggested by the values calculated by Krenchel [38] and Fisher et al. [39]. The values of R^2^ corresponding to fitting curves for both exponent fittings are presented in Table 3. The values of R^2^ corresponding to the ϕ^−1/5^ were around 0.95 for the microcellular nanocomposites prepared by WVIPS and 0.85 for the microcellular nanocomposites prepared by scCO_2_ dissolution foaming, whereas these values were 0.94 and 0.84 for the respective samples when the fitting curves were expressed as a function of ϕ^−1/3^.

The increase in the values of electrical conductivity, by increasing the gas volume percentage, can be explained by the reduction in GnP interparticle distance as a result of a reduction in the characteristic dimension of the continuous phase in the cellular structure through a more desirable re-orientation of graphene nanoparticles. Similar results have been reported by O. Maxian et al. [40], where their modeling of a percolation system, consisting of nanocomposites with carbon-based nanofillers, showed a decrease in terms of percolation threshold with increasing the porosity of nanocomposites. Gedler et al. [41] also presented the results of electrical conductivity enhancement with increasing the expansion ratio, with an optimum limit to reach maximum efficiency in augmenting the electrical conductivity.

## 5. Conclusions

In terms of GnP dispersion, the solution mixing step to produce microcellular nanocomposites by the WVIPS method was more effective when compared to the melt-mixing step used for the samples prepared by scCO_2_ dissolution. For the two series prepared using solution mixing (WVIPS), 15PSU and 25PSU, in the series with the lowest amount of polymer (15PSU), there was a better dispersion of graphene nanoplatelets, as indicated by X-ray diffraction. These changes could be directly related to the viscosity of the medium during mixing, as during melt-mixing the system possesses the highest viscosity, leading to a less effective dispersion of GnP, whereas solution mixing with the lowest amount of polymer (15PSU) had the lowest viscosity, resulting in the improved dispersion of the nanoparticles.

In terms of electrical conductivity, all the studied series of microcellular nanocomposites showed significant improvements with the amount of GnP, with the exception of the PSUCO_2_ samples, which showed limitations at GnP concentrations above 1 wt%. This could be related to a less effective dispersion of the nanoparticles at these concentrations. Among the microcellular nanocomposites prepared via WVIPS, the 15PSU samples showed significantly higher values of electrical conductivity, placing them among one of the highest reported values for this range of GnP content. The difference of three orders of magnitude for samples containing 10 wt% GnP between the 15PSU and 25PSU samples could be explained by the reduction in the interparticle distance, which could have resulted in assisting the formation of a conductive network. The tunnel conduction mechanism expressed the best fitting model for the values obtained in this study, representing a three-dimensional distribution of GnP. This indicates that high values of electrical conductivity could be obtained even with GnP stacks that were not fully exfoliated during mixing and foaming.

## Figures and Tables

**Table 1:** Density and intensity of the characteristic (002) diffraction peak of graphene for all the microcellular nanocomposites.

Foam Series	Foam Code	Density (g/cm^3^)	Intensity (a.u.)
15PSU	15PSU 1	0.347	233
	15PSU 2	0.355	537
	15PSU 5	0.415	812
	15PSU 10	0.534	2942
25PSU	25PSU 1	0.423	271
	25PSU 2	0.507	650
	25PSU 5	0.564	1786
	25PSU 10	0.598	3850
PSUCO_2_	PSUCO_2_ 0.1	0.435	134
	PSUCO_2_ 0.4	0.468	205
	PSUCO_2_ 0.7	0.467	497
	PSUCO_2_ 1.0	0.496	515
	PSUCO_2_ 1.5	0.561	739
	PSUCO_2_ 2.0	0.568	1009

**Table 2:** Electrical conductivity and corresponding corrected values of the PSU–GnP microcellular nanocomposites.

Foam Series	Foam Code	*V_g_* (%)	σ (S/m)	σ * (S/m)
15PSU	15PSU 1	72.1	2.31 × 10^−11^	1.20 × 10^−11^
	15PSU 2	71.6	3.43 × 10^−9^	1.72 × 10^−9^
	15PSU 5	67.3	2.38 × 10^−2^	1.11 × 10^−2^
	15PSU 10	58.8	8.17 × 10^−2^	8.17 × 10^−2^
25PSU	25PSU 1	66.1	1.63 × 10^−10^	8.93 × 10^−11^
	25PSU 2	59.4	1.73 × 10^−9^	8.79 × 10^−10^
	25PSU 5	55.5	1.16 × 10^−5^	1.16 × 10^−5^
	25PSU 10	53.9	6.08 × 10^−5^	6.08 × 10^−5^
PSUCO_2_	PSUCO_2_ 0.1	64.9	1.97 × 10^−12^	1.06 × 10^−12^
	PSUCO_2_ 0.4	62.3	9.55 × 10^−12^	5.71 × 10^−12^
	PSUCO_2_ 0.7	62.4	4.37 × 10^−11^	2.62 × 10^−11^
	PSUCO_2_ 1.0	60.2	2.56 × 10^−10^	1.53 × 10^−10^
	PSUCO_2_ 1.5	55.0	2.15 × 10^−10^	1.22 × 10^−10^
	PSUCO_2_ 2.0	54.6	8.12 × 10^−11^	5.02 × 10^−11^

σ *—corrected electrical conductivity.

**Table 3:** Coefficient of correlation for fitting curves corresponding to the corrected electrical conductivity (σ *) as a function of ϕ^−1/3^ and ϕ^−1/5^.

Foam Series	ϕ^−1/3^ R^2^	ϕ^−1/5^ R^2^
15PSU	0.95	0.95
25PSU	0.93	0.94
PSUCO_2_	0.84	0.85
